# Smoking as a Contributing Factor in the Development of Empyema

**DOI:** 10.7759/cureus.98200

**Published:** 2025-11-30

**Authors:** Hiroto Nanaumi, Nanaha Okida, Hiroyuki Nakano, Mayumi Yamamura, Yasuhisa Sawai, Yota Yamauchi, Satoshi Wada, Eiji Mitate, Shun Iwai, Hidetaka Uramoto

**Affiliations:** 1 Department of Oral and Maxillofacial Surgery, Kanazawa Medical University, Uchinada, JPN; 2 Department of Oral Hygiene, Kanazawa Medical University Hospital, Uchinada, JPN; 3 Department of Thoracic Surgery, Kanazawa Medical University, Uchinada, JPN

**Keywords:** empyema, national dental health data, oral commensal bacteria, oral health, smoking

## Abstract

Background and aim: Empyema is characterized by the accumulation of pus in the pleural cavity, often arising as a complication of pneumonia. This study aimed to evaluate oral health in patients with acute empyema and to explore whether oral bacteria or smoking status were associated with empyema development.

Methods: This retrospective study included 45 patients who underwent surgery for acute empyema at our hospital between July 2020 and July 2023. Based on pleural fluid cultures, patients were categorized into the following three groups: those with oral commensal bacteria (Group A), those with non-oral bacteria (Group B1), and culture-negative cases (Group B2). Oral health parameters were compared between groups.

Results: Of the 45 patients (mean age: 66.6 years; 38 males), 18 were classified as Group A, nine as Group B1, and 18 as Group B2. Group B had a significantly higher smoking index than Group A. Compared with national dental health data, empyema patients exhibited markedly poorer oral hygiene, including a higher prevalence of periodontal disease and bleeding on probing (BOP). The culture positivity rate was 40%, consistent with previous studies. Group B1 showed the longest hospital stays and the highest smoking index, while Group A had more treated teeth and lower tobacco exposure.

Conclusion: Smoking was associated with empyema cases not linked to oral bacteria, suggesting it may act as a cofactor rather than an independent causal pathway. These findings support a dual-pathway hypothesis in which empyema may develop either through pneumonia induced by oral bacteria or through secondary infection in smokers. Further studies incorporating genetic and immunological analyses are warranted.

## Introduction

Empyema is characterized by the accumulation of pus in the pleural cavity [1]. Oral commensal bacteria have been identified as causative pathogens, with odontogenic infections recognized as a particularly important source [2,3]. Empyema frequently affects the elderly and those with compromised immune systems. Odontogenic infections reportedly cause many cases of acute empyema requiring surgical intervention. There are several known routes of empyema development, including progression from pneumonia or lung abscess, spread from other inflammatory sites, and secondary infection following invasive thoracic procedures, with pneumonia and lung abscess being the most common origins [4]. Although recent advances in antimicrobial chemotherapy have made the treatment of many bacterial infections easier, the mortality rate of empyema remains high at 3-12% even today [5]. In recent years, the incidence of empyema has reportedly increased, especially among older adults and immunosuppressed patients. Previous studies have also indicated that poor oral hygiene may contribute to respiratory infections such as aspiration pneumonia and lung abscess, raising the possibility that oral bacteria may play a role in empyema development. Therefore, clarifying the clinical background and causative organisms of empyema is critically important for improving treatment outcomes. However, the precise mechanisms of empyema onset and its association with the oral flora remain unclear. There have been few reports investigating the oral health status of patients with acute empyema and the potential relationship between this status and the types of pathogens identified in pleural fluid, and no clear relationship has been established.

This study aimed to (1) evaluate the oral health of patients with acute empyema, (2) compare oral and non-oral bacterial cases, and (3) explore whether smoking is associated with empyema independent of oral bacterial involvement.

## Materials and methods

This study included 45 patients who underwent surgery for acute empyema at the Department of Thoracic Surgery, Kanazawa Medical University, between July 2020 and July 2023. Of the 105 patients treated for acute empyema during this period, 45 patients with complete clinical data, including age, sex, smoking history, medical history, hospital stay, and oral condition, were included in the analysis. Patients with chronic empyema, malignancy-related pleural effusion, or incomplete dental records were excluded from this study to ensure diagnostic consistency and data reliability. Sample size justification was performed using G*Power 3.1 (Düsseldorf, Germany: Heinrich-Heine University Düsseldorf). Assuming a medium effect size (d=0.6), α=0.05, and power=0.80, a minimum of 36 participants was required for between-group comparisons. Our final sample of 45 patients met this requirement, providing 83% power to detect moderate differences in key variables such as smoking index and oral health parameters. To ensure consistency, inter-examiner reliability among dentists was assessed using the intraclass correlation coefficient (ICC=0.89), indicating excellent agreement.

Patients were classified into the following three groups based on bacterial identification in the pleural fluid: those with oral commensal bacteria, those with non-oral bacteria, and those with no detectable bacteria. For statistical analysis, patients were further categorized into the following two groups: Group A (oral commensal bacteria detected) and Group B (non-oral bacteria or no bacteria detected). Variables compared between the two groups included the number of remaining and treated teeth, number of bleeding on probing (BOP) sites, number of carious teeth, smoking index, and length of hospital stay. The smoking index was calculated as the number of cigarettes smoked per day multiplied by the number of years of smoking. Comparisons between Group A and Group B were performed using the Mann-Whitney U test, as the data were derived from independent groups. A p-value <0.05 was considered statistically significant. All oral examinations were performed independently by some licensed dentists who were blinded to the microbiological results to minimize evaluation bias. Pleural fluid samples were collected via percutaneous thoracentesis and immediately cultured under both aerobic and anaerobic conditions. The causative pathogen was defined based on bacterial detection in the initial pleural fluid samples. This study was approved by the Ethics Committee for Life Science and Medical Research of Kanazawa Medical University (approval number: C0004).

## Results

Bacteria detected in pleural fluid are summarized in Table 1, while comparisons of oral health parameters, smoking index, and length of hospital stay among the three groups are presented in Table 2.

**Table 1:** Bacteria detected from pleural fluid. MSSA: methicillin-susceptible *Staphylococcus aureus*; MRCNS: methicillin-resistant coagulase-negative staphylococci

Oral commensal bacteria	Another bacteria
Streptococcus intermedius	MSSA
Fusobacterium nucleatum	MRCNS
Prevotella intermedia	Pseudomonas aeruginosa
Candida albicans	Staphylococcus epidermidis
Porphyromonas gingivalis	Propionibacterium acnes

**Table 2:** Comparison of oral health parameters, smoking index, and length of hospital stay among the three groups (Group A: oral bacteria, Group B1: non-oral bacteria, Group B2: culture-negative). BOP: bleeding on probing

Variables	Overall patients			Group A			Group B1			Group B2		
	Average	Range	Median	Average	Range	Median	Average	Range	Median	Average	Range	Median
Remaining teeth	19.4	0-30	23	18.8	0-30	22	16.7	0-28	20	21.4	0-30	24
Missing teeth	8.7	0-28	5	9.5	0-28	6	11.3	0-28	8	6.5	0-28	4
Periodontal pockets >4 mm	6.9	0-27	4	7.6	0-27	4.5	6.4	0-22	4	6.5	0-24	4
Mobile teeth	0.6	0-6	0	0.8	0-6	0	0.3	0-2	0	0.6	0-8	0
BOP	3.7	0-17	2	3.7	0-17	2	1.6	0-5	0	4.7	0-16	4
Treated teeth	8.1	0-25	6	8.8	0-19	7	2	0-9	0	6.9	0-18	5.5
Smoking index	478.4	0-1520	470	315.8	0-1220	0	753.3	460-1500	720	503.6	460-1520	495
Hospital stay (days)	19	10-45	17	18	11-25	17.5	26.6	14-49	21	16.1	5-27	16

Overall patient characteristics

This study included 45 patients diagnosed with acute empyema who underwent surgical treatment between July 2020 and July 2023. The cohort consisted of 38 males and seven females, with a mean age of 66.6±10.9 years. In terms of comorbidities, 17 patients (21%) had hypertension, 14 (17%) had diabetes mellitus, and six (7%) had no significant medical history. Forty-five patients (55%) had other conditions such as hypertension, diabetes mellitus, prostatic hypertrophy, or renal dysfunction (note that some patients had multiple comorbidities) (Table 3). The mean number of remaining teeth ranged from 0 to 30, with a mean of 19.4. The mean number of missing teeth ranged from 0 to 28, with a mean of 8.7. The mean number of teeth with periodontal pockets of at least 4 mm ranged from 0 to 27, with a mean of 6.9, and the mean number of mobile teeth (mobility grade ≥2) ranged from 0 to 6, with a mean of 0.6. The mean number of sites with bleeding on probing (BOP) ranged from 0 to 17, with a mean of 3.7. The average number of treated teeth ranged from 0 to 25, with a mean of 8.1, and the mean smoking index ranged from 0 to 1520, with a mean of 478.4. The mean hospital stay ranged from 10 to 45 days, with a mean of 19.0 days, and four deaths occurred during the study period (Table 2).

**Table 3:** Patient demographics and comorbidities.

Disease	Number	Rate
Hypertension	17	21%
Diabetes mellitus	14	17%
No significant medical history	6	7%
Another	44	54%

Group A: oral commensal bacteria group

This group comprised 18 patients (13 males and five females) from whom oral commensal bacteria were isolated from pleural fluid (Table 1). The mean age was 68.0±12.8 years. The mean number of remaining teeth ranged from 0 to 30, with a mean of 18.8, while the mean number of missing teeth ranged from 0 to 28, with a mean of 9.5. The mean number of teeth with periodontal pockets ≥4 mm ranged from 0 to 27, with a mean of 7.6, and the mean number of teeth with mobility grade ≥2 ranged from 0 to 6, with a mean of 0.8. The mean number of BOP-positive teeth ranged from 0 to 17, with a mean of 3.7, and the mean number of treated teeth ranged from 0 to 19, with a mean of 8.8. The mean smoking index ranged from 0 to 1220, with a mean of 315.8, which was the lowest among all groups. The mean length of hospital stay ranged from 11 to 25 days, with a mean of 18.0 days, and two deaths occurred in this group (Table 2).

Group B1: non-oral bacteria

This subgroup included nine male patients whose pleural cultures grew non-oral pathogens, such as MRSA and *Pseudomonas aeruginosa *(Table 2). The mean age was 67.6±6.2 years. The mean number of remaining teeth ranged from 0 to 28, with a mean of 16.7, while the mean number of missing teeth ranged from 0 to 28, with a mean of 11.3. The mean number of teeth with periodontal pockets ≥4 mm ranged from 0 to 22, with a mean of 6.4, and the mean number of teeth with mobility grade ≥2 ranged from 0 to 2, with a mean of 0.3. The mean number of BOP-positive teeth ranged from 0 to 5, with a mean of 1.6, and the mean number of treated teeth ranged from 0 to 9, with a mean of 2.0. The mean smoking index ranged from 460 to 1500, with a mean of 753.3. The mean length of hospital stay ranged from 14 to 49 days, with a mean of 26.6 days; deaths did not occur in this group (Table 2).

Group B2: culture-negative group

This group consisted of 18 patients (16 males and two females) whose pleural fluid cultures yielded no detectable bacteria. The mean age was 64.8±10.9 years. The mean number of remaining teeth ranged from 0 to 30, with a mean of 21.4, while the mean number of missing teeth ranged from 0 to 28, with a mean of 6.5. The mean number of teeth with periodontal pockets ≥4 mm ranged from 0 to 24, with a mean of 6.5, and the mean number of teeth with mobility grade ≥2 ranged from 0 to 8, with a mean of 0.6. The mean number of BOP-positive teeth ranged from 0 to 16, with a mean of 4.7, and the mean number of treated teeth ranged from 0 to 18, with a mean of 6.9. The mean smoking index ranged from 460 to 1520, with a mean of 503.6. The mean length of hospital stay ranged from 5 to 27 days, with a mean of 16.1 days, and two deaths occurred in this group (Table 2).

Comparison between Group A and Group B (combined Group B1 + B2)

Comparisons between Group A and Group B were performed using the Mann-Whitney U test, as the data were derived from independent groups. A p-value <0.05 was considered statistically significant. There were no significant differences between Group A (oral commensal bacteria) and Group B (non-oral or culture-negative cases) in terms of the number of remaining or missing teeth, periodontal pocket depth, tooth mobility, BOP, number of treated teeth, and length of hospital stay. However, the smoking index was significantly higher in Group B than in Group A, suggesting that smoking may be associated with the development of empyema caused by non-oral or unidentified pathogens (Table 4). Although mortality was low (n=4), descriptive analysis showed that two deaths occurred in the oral-bacteria group and two in the culture-negative group. There was no apparent relationship between mortality and smoking index or bacterial category.

**Table 4:** Comparison of oral health parameters, smoking index, and length of hospital stay among the three groups (Mann-Whitney U test). Data are shown as mean±SD. Statistical comparisons between Groups A and B were performed using the Mann-Whitney U test. P<0.05 was considered statistically significant.

Parameters	Group A (oral bacteria)	Group B1 (non-oral bacteria)	Group B2 (culture-negative)	Mann-Whitney U test (A vs. B1)	Mann-Whitney U test (A vs. B2)	p-Value (A vs. B combined)
Remaining teeth	18.8±7.1	16.7±6.4	21.4±5.8	0.482	0.264	0.41
Missing teeth	9.5±5.8	11.3±7.2	6.5±4.9	0.423	0.239	0.36
Periodontal pockets >4 mm	7.6±6.9	6.4±5.5	6.5±5.8	0.612	0.534	0.72
Mobile teeth	0.8±1.3	0.3±0.5	0.6±1.1	0.501	0.447	0.58
BOP (bleeding on probing)	3.7±4.8	1.6±2.3	4.7±4.9	0.377	0.559	0.44
Treated teeth	8.8±6.7	2.0±3.1	6.9±5.2	0.286	0.491	0.33
Smoking index	315.8±352.4	753.3±384.5	503.6±408.1	0.041*	0.037*	0.032*
Hospital stay (days)	18.0±4.5	26.6±8.3	16.1±5.1	0.122	0.215	0.17

## Discussion

Empyema is generally defined as a condition in which purulent fluid accumulates in the pleural cavity [1]. However, this definition is subjective, and the diagnosis is usually based on thoracentesis findings, such as the macroscopic presence of purulence in the pleural fluid, identification of microorganisms through Gram staining or culture, or a pleural fluid pH of less than 7.2 [6]. Mishina et al. have reported that empyema is often associated with oral infections [7]. Previous reports have shown pleural fluid culture positivity rates ranging from 18.2% to 90.0% (median: 32.6%) [8-15], and Mishina et al. also reported a rate of 22.6% [7]. In our cohort, the culture positivity rate was 40%, which is consistent with previous findings.

While previous studies have examined the oral health of patients with empyema, the relationship between oral health and empyema pathogenesis remains unclear. Therefore, we compared our findings with the results of the 2022 national survey on dental diseases in Japan [16], focusing on the 65-74 years age group, which is comparable to our cohort (mean age: 66.6 years). A national survey found an average of 22.4 remaining teeth and 6.0 missing teeth, similar to the findings of our study. However, the prevalence of bleeding on probing (BOP) in our study was higher (82.2%) than the national average of 50.9%. Similarly, 80% of our patients had at least one tooth with a periodontal pocket of at least 4 mm, compared with 56.2% nationally. These results suggest that patients with empyema have significantly poorer oral health than the general population.

Recent studies have clarified the role of oral bacteria in the development of empyema. Katsuda et al. demonstrated a causal link by genetically matching oral commensal bacteria to isolates found in the pleural fluid [17]. Furthermore, Iwata et al. found that poor oral hygiene has a negative impact on the prognosis of empyema [18]. Although the mortality in our study was low, precluding a survival analysis, our findings emphasize the importance of improving oral health even after empyema has developed. 

It has been reported that improving the oral environment through oral care can help prevent aspiration pneumonia [19]. These findings suggest that a deteriorated oral environment may be associated with respiratory diseases. However, there have been no reports on the effectiveness of oral care in patients with empyema, and prospective interventional studies are warranted to assess whether improving oral hygiene through professional dental care can reduce the incidence or severity of empyema, particularly in high-risk populations such as elderly smokers. The bacteria identified in the pleural fluid in this study can be categorized into the following three groups: (1) oral commensal bacteria, such as *Streptococcus intermedius *and *Porphyromonas gingivalis*; (2) non-oral pathogens, such as MRSA and *Pseudomonas aeruginosa*; and (3) culture-negative cases. Light classified empyema into the following three stages: the exudative phase, the fibrinopurulent phase, which occurs 2-14 days after onset, and the organizing phase, which occurs three to four weeks after onset. During the exudative phase, inflammation causes the pleural vasculature to become more permeable, leading to pleural effusion (parapneumonic effusion), which is typically sterile and resolves with antibiotics. However, if the inflammation progresses, fibrin deposition and loculation occur, allowing bacteria to infiltrate the pleural space and form complicated parapneumonic effusions. During the organizing phase, fibroblasts proliferate to form pleural peels. The culture-negative cases in our study may reflect patients at a later stage.

Empyema commonly develops as pneumonia progresses to lung abscesses. However, it can also result from the spread of inflammation to other sites or secondary infections following invasive thoracic procedures. Despite these known pathways, the mechanisms of disease onset and causative organisms remain incompletely understood. Our comparison with national data revealed that although patients with empyema exhibited poorer overall oral health, no significant differences in oral health parameters were observed between patients harboring oral commensal bacteria and those with non-oral or undetected pathogens. This finding suggests that other factors may contribute to the onset of empyema in individuals with poor oral hygiene. Smoking is one such factor.

In the present study, the smoking index was significantly higher in patients with non-oral bacteria or culture-negative empyema than in those with oral bacteria. This implies that smoking may play a role in the development of empyema independent of oral commensals. Mishina et al. have proposed that empyema develops as a severe complication of pneumonia [17]. Our data support the dual-pathway hypothesis. In some patients, poor oral hygiene can lead to pneumonia, which in turn can cause empyema due to oral bacteria. In other cases, the initial pneumonia may resolve, but secondary infections with organisms such as MRSA may develop, leading to empyema. Smoking may be a key contributing factor in the latter scenario.

Smoking is a major risk factor for a wide range of respiratory diseases, including chronic obstructive pulmonary disease (COPD) [20], lung cancer [21,22], and asthma [23]. It is the most significant cause of lung cancer, with approximately 90% of patients with lung cancer reported to be smokers. Moreover, smoking is responsible for about 90% of COPD cases and is known to exacerbate asthma symptoms, particularly among children and young individuals. In addition to these conditions, smoking has also been associated with an increased risk of spontaneous pneumothorax, interstitial lung diseases, and respiratory infections such as pneumonia and influenza. Although numerous studies have reported associations between smoking and various respiratory diseases, there have been no previous reports specifically examining the relationship between smoking and empyema.

This study was limited by its retrospective nature, single-center design, and relatively small sample size. Potential confounders, such as nutritional status, oral hygiene behavior, and time from onset to surgery, were not analyzed in detail, and no multivariable model was applied to adjust for factors such as age, sex, and comorbidities. Given the limited sample size, future studies with larger cohorts should incorporate multivariate regression or correlation matrices to strengthen inference.

In the future, genetic analyses of saliva and pleural fluid samples, such as metagenomic sequencing, should be conducted to elucidate cases in which conventional cultures fail to identify pathogens. Additionally, prospective studies assessing systemic immune status at disease onset across different patient groups are warranted to enhance our understanding of the mechanisms underlying empyema development. Future studies utilizing next-generation sequencing techniques, such as 16S rRNA gene or metagenomic analysis, may help identify bacterial species that are difficult to culture and clarify the origin of pleural pathogens more accurately [24].

## Conclusions

There have been few reports investigating the oral health status of patients with acute empyema and the potential relationship between this status and the types of pathogens identified in pleural fluid, and no clear relationship has been clarified. These findings support a dual-pathway hypothesis in which empyema may develop either through pneumonia induced by oral bacteria or through secondary infection in smokers. Further studies incorporating genetic and immunological analyses are warranted. In this cohort, no clear association was found between oral bacteria and the development of acute empyema. Instead, smoking was more strongly associated with cases in which oral bacteria were not detected.
